# Assemblage structure of ichthyoplankton in the NE Atlantic in spring under contrasting hydrographic conditions

**DOI:** 10.1038/s41598-019-44918-5

**Published:** 2019-06-14

**Authors:** J. M. Rodriguez

**Affiliations:** Instituto Español de Oceanografía. Centro Oceanográfico da Gijón, Avda. Principe de Asturias 70Bis, 33212 Gijón, Spain

**Keywords:** Ichthyology, Ichthyology, Ichthyology, Ichthyology, Ichthyology

## Abstract

The ichthyoplankton assemblage in the Atlantic region off the NW Iberian peninsula (AR) and in the southern Bay of Biscay (SBB) and the response of the larval fish species distribution to the relatively rapidly changing hydrographic conditions in spring 2010 were studied using two ichthyoplankton cruises. The SBB showed a more abundant and diverse ichthyoplankton assemblage than the AR, although the larval fish assemblage (LFA) was structured into on-shelf and off-shelf assemblages in both regions. Inter-sampling variability related to downwelling/upwelling regimes was observed in the cross-shelf assemblage distribution in the SBB but not in the AR. This suggests that LFA distributions in the area of this study are good indicators of downwelling and weak coastal upwelling regimes but not of relatively strong coastal upwelling or upwelling filaments. Although depth was identified by multivariate analyses as being the most important variable explaining larval fish species distributions, a shelf-breakfront in the SBB and the surface offshore (onshore) flows associated with coastal downwelling (upwelling) in the AR seems to have been key in defining and maintaining assemblage boundaries. Results of this study should also encourage marine research institutions to use routine ichthyoplankton sampling to monitor fish communities and their responses to global warming.

## Introduction

The composition of the ichthyoplankton assemblage found in an area during a study depends on the adult fish species that are reproducing at the time of the study, while its structure will be determined by the abundance of these species and by the mortality rates of their offspring, which are in turn influenced by the hydrographic processes involved in the horizontal distribution of fish eggs and larvae: fronts, eddies, marine currents, Ekman transport and upwelling filaments, among other mesoscale hydrographic features^1–4^. Biological factors, such as the spawning location, the spawning strategy, and larval behaviour, also play a role in ichthyoplankton distribution and survival^3,5,6^ and consequently in defining the composition and structure of the ichthyoplankton assemblage.

**Figure 1:**
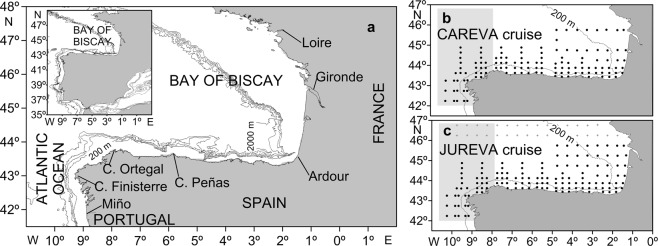
(**a**) Study area including the Atlantic region (shadow area in **b**,**c**) and the southern Bay of Biscay, the southwesternmost border between them is Cape Ortegal. (**b**) Sampling stations for the CAREVA cruise. (**c**) Sampling stations for the JUREVA cruise: (•) common stations sampled during both cruises and used in comparative analyses; (+) stations for which hydrogrpahic and biological features and ichthyoplankton distributions were described and depicted; (△) stations only included in the larval fish assemblage composition.

There is little information on the ichthyoplankton assemblage and on its response to environmental variables in the area of this study, which includes two geographic regions, the Atlantic region off the NW Iberian peninsula (AR) and the southern Bay of Biscay (SBB), located in the NE Atlantic (Fig. 1). Most of the ichthyoplankton studies carried out in this area had the aim of generating fishery-independent stock assessments. In fact, the CAREVA 1003 and the JUREVA 1004 cruises (from now on the CC and the JC, respectively) involved in this study are triennial cruises that occur within a large sampling scheme, covering a large sampling area that extends from approximately 42°N to approximately 60°N along the European coasts and that includes several countries. Both cruises, carried out at the spawning peaks of *Scomber scombrus* (the CC) and *Trachurus trachurus* (the JC), are conducted to obtain data on early life stages and on adults of these species for the international evaluation of their spawning stock biomass in the NE Atlantic. Consequently, most of these studies have focused on commercially important fish species, generally a single species, mainly *Sardina pilchardus*^7,8^, or a few species^9^. Studies dealing with the ichthyoplankton assemblage are scarce and did not cover the whole area included in this study^10–13^. Thus, this is the first study to use opportunistic ichthyoplankton sampling to address the ichthyoplankton assemblage in the AR and the SBB and its response to environmental variables. The aims of this study were to identify the composition and structure of the ichthyoplankton assemblage in the region and to analyse the role of the environmental variables depth, sea surface temperature, sea surface salinity, water column stratification and mesozooplankton biomass and the hydrographic features found during the study in shaping the horizontal structure of the larval fish assemblage (LFA) in the region, in spring, under contrasting environmental conditions. I also hypothesized that the cross-shelf distribution of larval fish assemblages (LFAs) is a good indicator of the upwelling/downwelling regimes in spring in the area of study. The use of fish larvae as tracers of hydrographic processes has already been proposed elsewhere, e.g. in the NW African upwelling region^2^, the Taiwan strait^14^, the Sicilian channel^15^ and the Atlantic region in our area of study^11^. Moreover, in the context of global change, ichthyoplankton studies could be used to evaluate the changes that are expected to occur in the composition and structure of the fish community in the area of study as consequence of global warming. This would have special relevance in the Bay of Biscay, where, as consequence of the warming of the Iberian Poleward Current (described below), a pronounced increase in tropical fish species has been recorded in recent decades^16^. These species move poleward with warming trends to remain within suitable “climate envelopes“^17^. However, for these species to be able to establish in the region, they must reproduce successfully and their offspring must survive in the new area^18^ (and references herein). Other species can change their spawning areas^9^. In both cases, changes in fish species composition or in their spawning areas would be reflected in the composition and structure of the larval fish assemblage, and these changes could be evaluated through ichthyoplankton studies. According to Koslow and Wright^19^ “ichthyoplankton surveys provide a relatively low-cost, efficient means to monitor marine fish populations and communities”.

## Materials and Methods

### The area of study

The AR constitutes the northern limit of the large eastern north Atlantic coastal upwelling system^20^, while the Bay of Biscay is a Large Marine Ecosystem^21^, and the southwesternmost border between the two regions is considered to be Cape Ortegal (Fig. 1a)^22^. The shelf is narrow along the Spanish coast, widening from south to north along the French coast (Fig. 1a). Along this coast, two relatively large rivers (the Loire and Gironde) and another smaller river (the Ardour) discharge into the Bay of Biscay, forming large shelf plumes^23^. Along the north-western and northern coasts of Spain, the rivers are small, with small outflows that do not form large coastal plumes^23^.

The hydrography of the study area is highly influenced by seasonal factors. In autumn and winter, southerly and westerly winds, which are favourable to coastal downwelling, predominate, and the surface current over the shelf flows poleward^24,25^. During these seasons, the shelf-slope circulation is dominated by the Iberian Poleward Current, a geotropically balanced poleward flow^26,27^. This current flows along the continental slope of the western Iberian Peninsula, enters the Bay of Biscay^24,28^ and eventually reaches approximately 48°N on the French Shelf^29^. The Iberian Poleward Current is an unstable flow that transports warm and salty waters and induces the formation of convergent fronts separating salty oceanic water from fresher coastal waters and eddies^27,30^. In the SBB, these eddies, shed from the slope into the Bay, are named SWODDIES (anticyclonic eddies of slope water), have a lifespan of approximately one year and drift westward at a speed of 2 cm^−1^ ^31^. The surface layer of the oceanic region may be occupied either by the warmer and saltier eastern North Atlantic central water of subtropical origin transported by the Iberian Poleward Current or by the colder and fresher eastern North Atlantic central water of subpolar origin^29^.

During spring and summer, strong wind-driven coastal upwelling events are frequent in the AR^20^. In the SBB, coastal upwelling events are weak and less frequent^22,32^. Topographic forcing, mainly induced by large capes, may lead to the formation of upwelling filaments^33^, extensions of coastally upwelled water that may reach hundreds of kilometres offshore^34^. Cape Ortegal and mainly Cape Finisterre have associated large upwelling filaments^33^, while Cape Peñas has associated a minor upwelling filament^22^. The filament associated to Cape Finisterre is a recurrent feature that may extend up to 200 km offshore^20,33^. During upwelling, the surface current over the shelf flows equatorward^24,35^. The transition from autumn-winter downwelling to spring-summer upwelling conditions occurs around April^36^.

### Sampling and data collection

This study is based on hydrographic data and zooplankton samples collected during the CC and the JC carried out on board of the research vessel Cornide de Saavedra from March 15 to April 6 (the CC) and from April 15 to May 16 of 2010 (the JC) in an area extending from the mouth of the Miño river to the latitude of 46.77°N (Fig. 1a). A grid of 168 stations was intended to be sampled. This grid was sampled during the JC (Fig. 1c). During the CC, because of bad weather, only 126 stations out of 168 were sampled (Fig. 1b). The CC and the JC are triennial ichthyoplankton cruises carried out by the Spanish Institute of Oceanography within the Spanish national program of collection, management and use of data for scientific advice regarding the common fisheries policy of the European Union.

At each sampling station, vertical profiles of conductivity and temperature were recorded with a SeaBird 25 CTD (conductivity, temperature, depth) probe to 200 m depth or to 5 m above the bottom at shallower stations.

Daily values of the upwelling index for the sampling periods were provided by the Spanish Institute of Oceanography (http://www.indicedeafloramiento.ieo.es/). These values were calculated using the sea level pressure, obtained from the Fleet Numerical Meteorology and Oceanography Center (www.usno.navy.mil/FNMOC), to determine the geostrophic wind in a cell centred at 44°N, 9°W^37^. Positive values of the upwelling index correspond to upwelling, while negative values correspond to downwelling.

Zooplankton samples were collected with a bongo net of 40 cm mouth diameter and 250 µm mesh size. Each net mouth was equipped with a General Oceanics flowmeter to measure the volume of water filtered. The net was also equipped with a Seabird 37 CTD probe to measure the haul depth and to obtain a haul profile to control the quality of the sampling. Ichthyoplankton tows were oblique, from 200 m depth (or from ~5 m above the bottom at shallower stations) to the surface. Fish eggs from one of the samples of the bongo net were pre-sorted on board using the “spray method”^38^. After that, samples were immediately preserved in a 4% solution of buffered formalin and seawater.

In the laboratory, unsorted eggs were separated from the samples pre-sorted on board, and fish larvae were sorted from the other bongo samples. All fish larvae were identified to the lowest taxonomic level possible, while fish eggs belonging only to *S*. *pilchardus*, *S*. *scombrus* and *Engraulis encrasicolus* were identified to the species level. Fish egg and larval counts were standardized to number 10 m^−2^ of sea surface (abundance). Larval fish taxa were grouped into two categories based on the habitat of adult fishes and on the region where they reproduce: neritic, in which adult fishes roughly inhabit and reproduce over the shelf, and oceanic, in which adult fishes roughly inhabit and reproduce off the shelfbreak.

After sorting the ichthyoplankton, samples were used to determine the mesozooplankton biomass. The methodology employed was as follows. First, the preservative liquid was eliminated, and the samples were washed to eliminate formalin residue. After that, the samples were filtered through sieves of 2000 and 200 µm mesh size. Zooplankton organisms >2000 µm, those retained by the 2000 µm sieve, were eliminated. The fraction of the sample retained by the 200 µm mesh sieve was filtered through a tared Whatman GFF filter, previously dried in an oven at 60 °C to constant weight. The dry weight of the mesozooplankton was standardized to mg m^−2^ of sea surface (mesozooplankton biomass).

In the evaluation of the composition of the LFA, all the stations sampled during the JC were included. This gives us an idea of the composition of the LFA in the AR and in the SBB during spring. However, hydrographic and biological features and ichthyoplankton distributions during this cruise were described and depicted for the entire French shelf region sampled (Fig. 1b). Finally, for comparisons between the CC and the JC, only the common area sampled during both cruises was considered. This was done to exclude the area not sampled during the CC, where different variables may affect the ichthyoplankton assemblage.

### Data analysis

#### Univariate analyses

The difference in water density between 200 m (or 5 m above the bottom at shallow stations) and 5 m depth was used as an index of water column stratification^39,40^.

Larval fish diversity was calculated for each station using the Shannon–Wiener diversity index.

Pairwise comparisons of variables were conducted with Student’s t-test for independent samples. Differences in ichthyoplankton abundances, larval fish taxon richness and diversity between cruises (the CC and the JC) and cruises-regions combined (AR sampled during the CC and the JC and SBB sampled during the CC and the JC) were tested with two-way ANOVA followed, when necessary, by a post hoc Student-Newman-Keuls test. The relationships between variables were tested with Pearson’s correlation coefficient.

Prior to the statistical analyses, variables were tested for normality (Kolmogorov–Smirnov test) and homogeneity of variances (Levene’s test). As a result of these tests, ichthyoplankton abundances and the environmental variable depth were log_10_ transformed before the analyses.

#### Multivariate analyses

Differences in LFA structure between the CC and the JC and between the CC and the JC in the AR and the SBB were assessed with two-way permutational multivariate ANOVA (PERMANOVA) for unbalanced designs^41^. PERMANOVA was carried out with a Bray-Curtis similarity matrix generated from the fourth root-transformed data of larval fish taxon abundances from both cruises combined. The data were transformed to down-weight the influence of the more abundant taxa^42,43^. Because significant differences in assemblage structure between cruises and regions were detected (see ‘Results’), in a second step, cluster analysis was used to identify LFAs within each region for each cruise. Cluster analysis was performed on four (one per region and cruise) Bray-Curtis similarity matrices generated from the fourth root-transformed data of larval fish taxon abundances. Hierarchical agglomerative clustering with group-average linking carried out with the similarity matrices was used to delineate assemblages. Dendrograms were cut off at arbitrary levels to produce ecologically interpretable clusters^42,44^. The adequacy of the classification analysis was checked by superimposing cluster results on non-metric multidimensional scaling (nMDS) ordination plots generated from the same similarity matrices^43–45^. A 2-dimensional ordination approach was adopted because stress levels (0.13–0.16) were acceptably low^43,45^. Rare taxa (those found at fewer than 5% of the sampling stations in each region during each cruise) and sampling stations with 0 larvae were not included in the cluster analyses or the nMDS ordination^42,43^. The differences in structure among LFAs, as delineated by cluster analyses and confirmed by nMDS ordination, were assessed with PERMANOVA.

The relationships between environmental variables and larval fish taxa were assessed with canonical correspondence analysis (CCA). The environmental variables considered in this study were bottom depth at each station (depth), temperature in °C at 5 m depth (sea surface temperature), salinity at 5 m depth (sea surface salinity), water column stratification and mesozooplankton biomass. The collinearity among explanatory variables was assessed with Pearson’s correlation coefficient. As above, rare taxa (those present at <5% of the stations) and samples with 0 larvae were not included in the analysis. Rare taxa may have a large influence on the analysis^46^. Larval abundances were log_e_(x + 1) transformed before analysis. Forward stepwise selection of explanatory variables was carried out using the Monte Carlo permutation test. This process also allows explanatory variables to be ranked according to their importance in taxon ordination^46,47^. However, informally, the importance of a variable is also indicated by the length of its corresponding environmental arrow in the CCA ordination plots^47^. Only those variables significant in explaining taxon distribution patterns were included in the analyses. Canonical axes were also tested for significance^46,48^.

Only larvae identified to at least the genus level were included in the multivariate analyses. Additionally, in all statistical analyses, the level of statistical significance (α) was set at 0.05.

Diversity index calculation, PERMANOVA, cluster analysis and MDS ordination were performed with PRIMER v7^41,43^. Correlation, Student’s t-test and ANOVA analyses were performed with IBM SPSS Statistics version 19, while the CCA ordination was conducted using CANOCO version 4.56^46^.

### Ethical approval

This article does not contain any studies with human participants or animals performed by the author.

## Results

### Environmental variables

**Figure 2:**
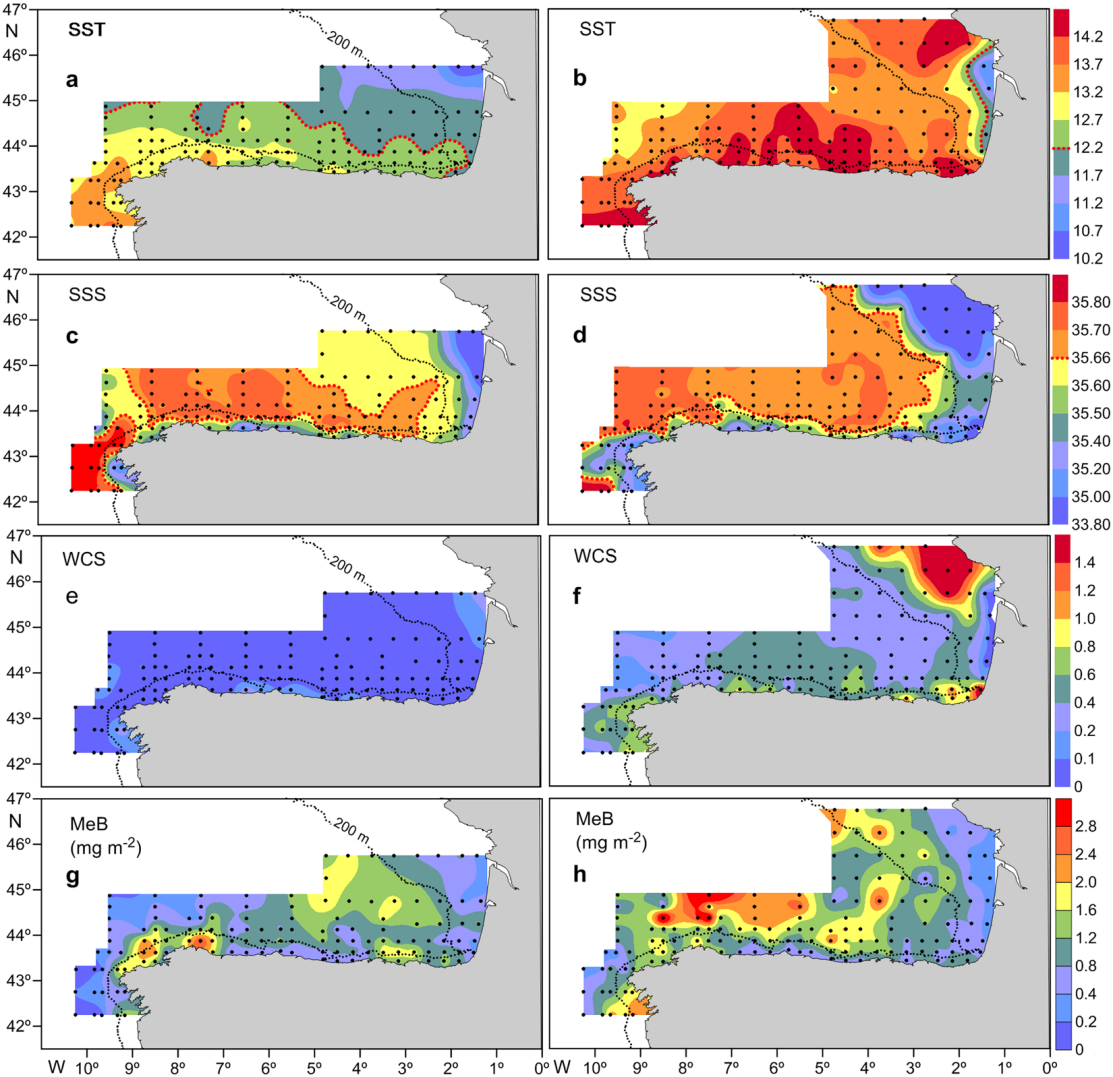
Horizontal distribution of: (**a**) Sea surface temperature (SST, °C at 5 m depth) during the CAREVA cruise (CC). (**b**) SST during the JUREVA cruise (JC). (**c**) Sea surface salinity (SSS, salinity at 5 m depth) during the CC. (**d**) SSS during the JC. (**e**) Water column stratification (WCS, differences in water density between 200 m or 5 m above the bottom and 5 m depth) during the CC. (**f**) WCS during the JC. (**g**) Mesozooplankton biomass (MeB, mg m^−2^) during the CC. (**h**) MeB during the JC. The dotted redlines in a and b indicate the 12.2 °C isotherm and in c, and d the 35.66 isohaline.

During the CC, there was a tongue of warm (>13.2 °C) and salty (>35.80) surface water extending along the NW Spain coast (Fig. 2a,c), which was reduced to a patch located in the southwesternmost part of the study area during the JC (Fig. 2b,d). In the SBB, the sea surface temperature showed a roughly zonal distribution during the CC (Fig. 2a) and an irregular distribution during the JC, with a region of relatively cold water along the French coast (Fig. 2b). During both cruises, the coastal waters were fresher than the slope and oceanic surface waters, while most of the slope and oceanic surface waters were relatively warm (>12.2 °C)(Fig. 2a,b) and salty (>36.6) (Fig. 2c,d). The coastal, fresher waters showed a wider offshore distribution, mainly along the French coast, during the JC (Fig. 2d). When he presence of fresh water filaments westward of the main capes of the Iberian Peninsula, Cape Peñas, Cape Ortegal and Cape Finisterre was noteworthy, and the largest one, that of Cape Finisterre, affected most of the AR (Fig. 2d). The sea surface temperature was highest during the JC (Student’s t-test, p < 0.01). However, there was no difference in sea surface salinity between the two cruises (Student’s t-test, p > 0.05).

The water column was almost completely mixed during the CC (Fig. 2e) and slightly more stratified during the JC, mainly in coastal areas (Fig. 2f). The values representing water column stratification were significantly higher during the JC (Student’s t-test, p < 0.01). This variable was strongly correlated with sea surface salinity, with Pearson’s correlation coefficients of r = 0.73 for the CC and r = 0.81 for the JC (p < 0.01 in both cases). The Pearson’s correlation coefficients between water column stratification and sea surface temperature were r = 0.03 (p > 0.5) for the CC and r = 0.47 (p < 0.01) for the JC.

**Figure 3:**
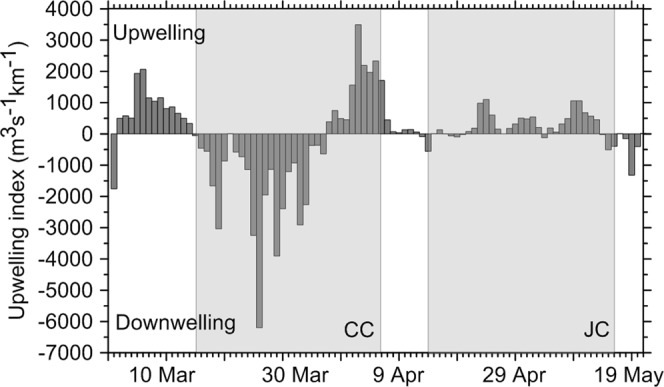
Daily upwelling index calculated at 44°N, 9°W, from the 1 March to 20 May 2010. Shaded regions denote the time of the cruises (CC, CAREVA cruise; JC, JUREVA cruise).

Hydrographic conditions of relatively strong or strong downwelling prevailed during most of the CC, except at its end, when a relatively strong upwelling event was recorded (Fig. 3). However, weak upwelling prevailed during most of the JC (Fig. 3). The average values of the upwelling index were −856.3 for the CC and 187.9 for the JC (negative values correspond to downwelling, while positive values correspond to upwelling).

Mesozooplankton biomass values were between 0.05 and 2.94 mg m^−2^, with a mean of 0.96 (±5.86 SD) mg m^−2^, during the CC and ranged from 0.17 to 4.68 mg m^−2^, with an average of 1.25 (±7.43 SD) mg m^−2^, during the JC. Mesozooplankton biomass values were significantly higher during the JC (Student’s t-test, p < 0.01). This variable showed irregular distribution patterns during both cruises (Fig. 2g,h), drawing attention to the relatively high values of mesozooplankton biomass recorded in the oceanic region of the SBB during the JC (Fig. 2h).

### The ichthyoplankton assemblage

A total of 62,928 fish eggs and 13,231 fish larvae were captured during the CC, and 32, 405 fish eggs and 27,684 larvae were collected during the JC. The most abundant fish egg species were *S*. *scombrus* during the CC and *S*. *pilchardus* during the JC, accounting for 67.18% and 38.00% of the total fish egg abundances, respectively. Combining both cruises, the most abundant fish egg species was *S*. *scombrus* (46.15% of the total fish egg abundances), followed by *S*. *pilchardus* (19.70%) and *E*. *encrasicolus* (3.90%).

**Figure 4:**
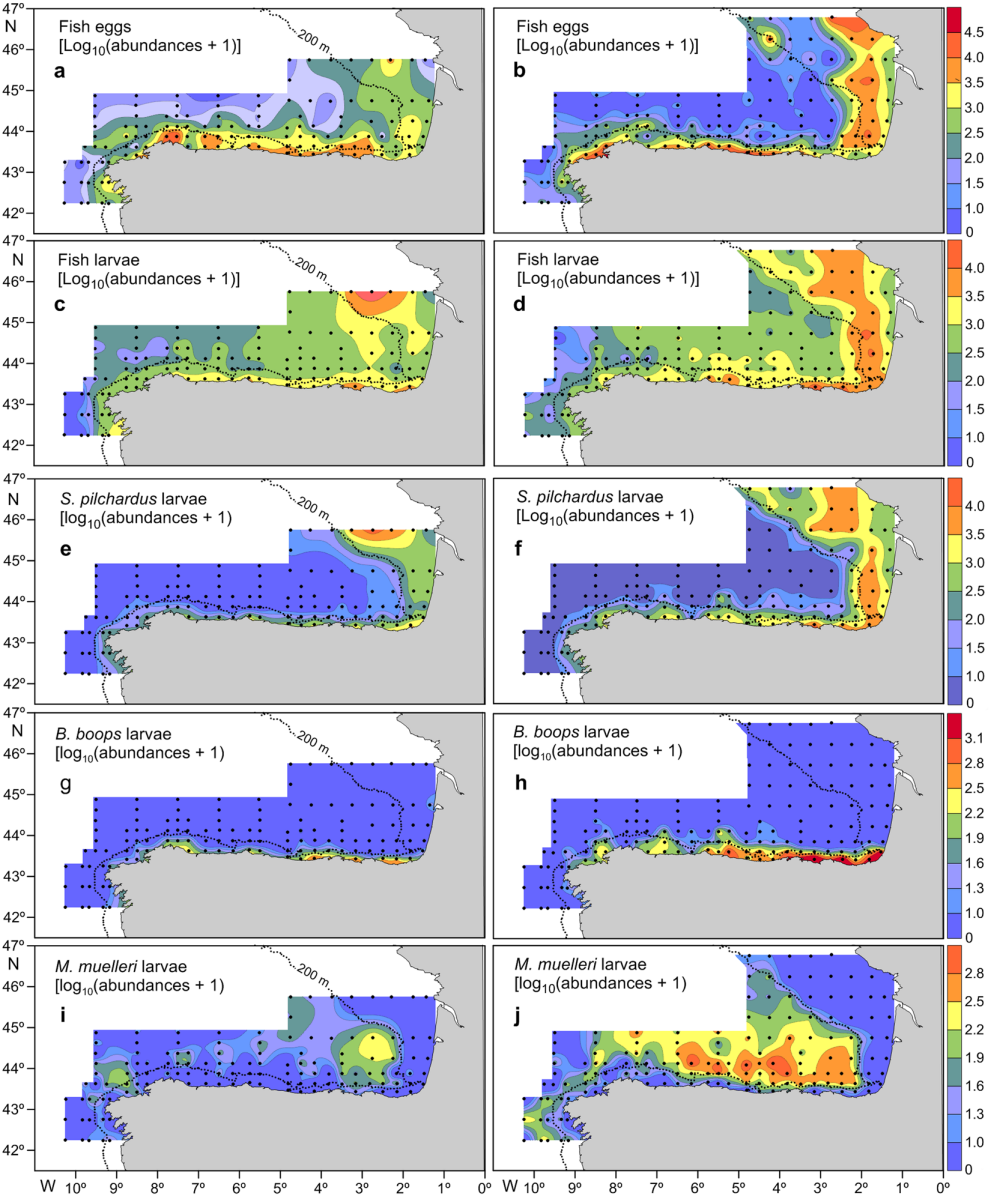
Horizontal distribution of: (**a**) Fish eggs during the CAREVA cruise (CC); (**b**) Fish eggs during the JUREVA cruise (JC); (**c**) Fish larvae during the CC; (**d**) Fish larvae during the JC; (**e**) *Sardina pilchardus* larvae during the CC; (**f**) *S*. *pilchardus* larvae during the JC; (**g**) *Boops boops* larvae during the CC; (**h**) *B*. *boops* larvae during the JC; (**i**) *Maurolicus muelleri* larvae during the CC; (**j**) *M*. *muelleri* larvae during the JC.

Fish egg abundances ranged from 9.72 to 181,969.72 eggs 10 m^−2^, with a mean of 4462.21 (±8833.10 SD) eggs 10 m^−2^, during the CC and from 0 to 46,194.54 eggs 10 m^−2^, with a mean of 2056.32 (±8833.10 SD) eggs 10 m^−2^, during the JC. Differences in fish egg abundances were significant between cruises, with a higher abundance during the CC, and among cruises-regions combined (Table 1). When the two cruises were combined, fish egg abundances were significantly higher in the SBB (Student’s t-test, p < 0.01). The patterns of the horizontal distribution of fish eggs were similar during the two cruises, with higher abundances recorded close to the coast (Fig. 4a,b), although relatively high abundances were recorded in the oceanic region during the CC, mainly in the SBB (Fig. 4a).

**Table 1:** Results of the two-way ANOVA used to test for the differences in ichthyoplankton abundance, larval fish species richness and larval fish diversity among cruises (CAREVA cruise: CC and JUREVA cruise: JC) and cruises-regions (ARCC: Atlantic region sampled during the Careva cruise; ARJC: Atlantic region sampled during the Jureva cruise; SBBCC: southern Bay of Biscay region sampled during the Careva cruise and SBBJC: southern Bay of Biscay region sampled during the Jureva cruise).

Source of variation	SS	df	*F*	p
**Fish egg abundances**				
Cruise	11.72	1	9.47	<0.01
Cruise-Region	9.75	1	7.88	<0.01
Cruise:Cruise-Region	1.16	1	0.94	NS
Error	319.34	258		
SNK test: SBBCC > ARCC > ARJC = SBBJC				
**Larval fish abundances**				
Cruise	0.23	1	0.67	NS
Cruise-Region	19.34	1	56.62	<0.01
Cruise:Cruise-Region	0.10	1	0.28	NS
Error	88.11	258		
SNK test: SBBJC = SBBCC > ARCC = ARJC				
**Larval fish species richness**				
Cruise	9.10	1	0.22	NS
Cruise-Region	1148.90	1	28.19	<0.01
Cruise:Cruise-Region	6.10	1	0.15	NS
Error	10516.00	258		
SNK test: SBBCC = SBBJC > ARCC = ARJC				
**Larval fish diversity**				
Cruise	1.00	1	3.15	<0.01
Cruise-Region	5.27	1	16.66	<0.01
Cruise:Cruise-Region	5.27	1	0.06	NS
Error	81.51	258		
SNK test: SBBCC = SBBJC > ARCC > ARJC				

Results of the Student-Newman-Keuls (SNK) post hoc test for cruise-regions (see text for the acronyms of cruise-region) are also included. (NS: not significant).

A total of 76 taxa (66 species, 3 genera and 7 families) of fish larvae in 32 families were recorded during the CC, while during the JC, 97 taxa (88 species, 3 genera and 6 families) of fish larvae from 38 families were collected (Table 2). When the two cruises were combined, 104 taxa (93 species, 3 genera and 8 families) of fish larvae from 39 families were recorded (Table 2). The most abundant species during both cruises was *S*. *pilchardus*. The most ubiquitous species were *S*. *scombrus* during the CC and *Maurolicus muelleri* during the JC (Table 2). When the two cruises were combined, the most abundant and most ubiquitous species were *S pilchardus* and *M*. *muelleri*, respectively (Table 2). The relatively high number of oceanic fish species recorded and the ubiquity of some of them are remarkable (Table 2).

**Table 2:** Alphabetical list of larval fish families collected during the CAREVA 0310 (CC).

Family and species	Code	Origin	CC		JC		CC + JC	
			RA	%Occ	RA	%Occ	RA	%Occ
**Ammodytidae**								
*Ammodytes tobianus*		N	0.05	4.0	0.02	2.4	0.03	3.1
*Gymnammodytes semisquamatus*	*Gs*	N	0.70	16.7	0.02	1.8	0.23	8.2
*Hyperoplus lanceolatus*	*Hl*	N	0.53	12.7	0.06	6.5	0.20	9.2
**Argentinidae**								
*Argentina sphyraena*	*As*	N	0.08	7.1	0.10	10.7	0.09	9.2
**Bathilagidae**								3.4
*Bathilaguss bericoides*	*Bb*	Oc	0.78	36.5			0.24	11.6
Unidentified spp		Oc	0.09	4.8	0.02	2.4	0.04	
**Blennidae**								
*Coryphoblennius galerita*		N			<0.01	0.6	<0.01	0.3
*Lipophrys pholis*		N			<0.01	0.6	<0.01	0.3
*Lipophrys trigloides*		N			0.01	1.2	0.01	0.7
*Parablennius gattorugine*	*Pg*	N	0.10	6.3	0.04	3.6	0.06	4.8
*Parablennius pilicornis*	*Pp*	N			0.13	9.5	0.11	5.4
*Parablennius sanguinolentus*	*Ps*	N	0.06	4.8	0.04	3.6	0.03	4.1
**Bothidae**								
*Arnoglossus imperialis*	*Ai*	N	1.48	23.8	0.07	6.0	0.51	13.6
*Arnoglossus laterna*	*Al*	N	0.91	25.4	1.56	27.4	1.36	26.5
*Arnoglossus thori*	*At*	N	0.31	12.7	0.41	19.6	0.38	16.7
*Zeugopterus punctatus*	*Zp*	N	0.20	5.6	0.01	2.4	0.07	3.7
**Callionymidae**								
*Callionymus* spp	*C*spp	N	4.06	49.2	5.89	52.4	5.33	51.0
**Carangidae**								
*Decapterus punctatus*		N			0.00	0.6	<0.01	0.3
*Trachurus trachurus*	Tt	N	1.70	33.3	1.57	33.3	1.61	33.3
**Cepolidae**								
*Cepola rubescens*		N			0.03	1.2	0.02	0.7
**Clupeidae**								
*Sardina pilchardus*	Sp	N	39.04	52.4	45.21	60.1	43.32	56.8
**Engraulidae**								
*Engraulis encrasicolus*	*Ee*	N			3.66	11.9	2.539	6.8
**Gadidae**								
*Gadiculus argenteus*	*Ga*	Oc	0.42	14.3	0.01	1.8	0.14	7.1
*Micromesistius poutassou*	*Mp*	Oc	6.66	54.0	0.04	2.4	2.06	24.5
*Merlangius merlangus*		N	0.05	4.0	0.02	1.2	0.03	2.4
*Pollachius pollachius*	*Ppo*	N	0.10	5.6			0.03	2.4
*Trisopterus luscus*	*Tl*	N	0.58	28.6	0.05	4.8	0.21	15.0
*Trisopterus minutus*	*Tm*	N	1.70	25.4	0.18	13.1	0.65	18.4
Gadidae sp 1					0.01	0.6	0.01	0.3
Gadidae sp 6					0.00	0.6	<0.01	0.3
Gadidae sp 7			0.02	1.6			0.01	0.7
Unidentified spp			0.06	4.8	0.01	1.2	0.03	
Gobiesocidae								
*Lepadogaster lepadogaster*		N			<0.01	0.6	<0.01	0.3
**Gobiidae**								
*Crystallogobius linearis*		N	0.03	3.2	<0.01	0.6	0.01	1.7
*Gobius paganellus*		N			0.03	3.6	0.02	2.0
*Lebetus guilleti*	*Lg*	N	0.40	17.5	0.35	19.0	0.37	18.4
*Pomatoschistus marmoratus*	*Pm*	N	0.06	4.8	0.09	6.5	0.08	5.8
*Pomatoschistus microps*	*Pmi*	N	0.39	11.9	0.44	14.9	0.48	13.6
*Pomatoschistus minutus*	*Pmin*	N	0.88	27.0	0.66	26.8	0.74	26.9
*Pomatoschistus pictus*		N			0.02	1.2	0.01	0.7
*Pomatoschistus* spp	*P*spp	N	1.09	26.2	1.67	26.2	1.49	26.2
Unidentified spp		N	0.39	20.6	0.90	29.8	0.74	
**Gonostomatidae**								
*Cyclothone braueri*		Oc			<0.01	0.6	<0.01	0.3
**Labridae**								
*Coris julis*		N			0.01	0.6	0.01	0.3
*Ctenolabrus rupestris*	*Cr*	N	0.05	4.0	0.40	16.7	0.29	11.2
*Labrus bergylta*	*Lb*	N	0.20	9.5	0.15	9.5	0.17	9.5
*Labrus bimaculatus*	*Lbi*	N	0.31	17.5	0.07	8.9	0.15	12.6
*Symphodus melops*	*Sm*	N			0.04	3.0	0.03	7.1
**Liparidae**								
*Liparis* sp		N	0.01	0.8	0.01	0.6	0.01	0.7
**Lotidae**								
*Antonogadus macrophthalmus*		Oc	0.06	4.0			0.01	1.7
*Gaidropsarus mediterraneus*	*Gm*	N	1.17	31.7	0.10	9.5	0.43	19.0
*Gaidropsarus vulgaris*	*Gv*	N	1.51	33.3	0.20	13.1	0.60	21.8
**Macrouridae**								
Macrouridae sp 1		Oc			0.01	0.6	0.01	0.3
**Merlucciidae**								
*Merluccius merluccius*	*Mm*	N	0.53	18.3	0.22	16.1	0.32	17.0
**Moronidae**								
*Dicentrarchus labrax*	*Dl*	N	0.08	7.1	0.03	3.6	0.05	5.1
**Microstomidae**								
*Nansenia groenlandica*		Oc	0.04	4.0	0.02	3.0	0.03	3.4
Mugilidae								1.4
*Mugil cephalus?* Mugilidae sp 1		N			0.01	1.2	0.01	0.7
Unidentified spp			0.02	1.59	0.01	1.2		
**Mullidae**								
*Mullus surmuletus*	*Ms*	N			0.16	10.1	0.11	5.8
**Myctophidae**								
*Benthosema glaciale*	*Bg*		3.85	48.4	2.82	56.0	3.14	52.7
*Cerastocopelus maderensis*		Oc			<0.01	0.6	<0.01	0.3
*Lampanyctus crocodilus*	*Lc*	Oc	0.15	12.7	0.31	22.6	0.26	18.4
*Lampanyctus* sp 1		Oc			<0.01	0.6	<0.01	0.3
*Myctophum punctatum*	*Mpu*	Oc	0.54	29.4	1.04	50.0	0.89	41.2
*Notoscopelus resplendens*	*Nr*	Oc	2.31	52.4	1.44	52.4	1.71	52.4
Unidentified spp			0.09	4.8	0.01	1.2	0.04	
**Paralepididae**								0.7
*Lestidiops jayakari*	*Lj*	Oc	0.08	7.1	0.45	32.7	0.34	21.8
*Paralepis coregonoides*	*Pc*	Oc	1.78	39.7	1.54	49.4	1.62	45.2
*Paralepis sphiraenoides*		Oc			0.01	1.2	0.01	0.7
Unidentified spp		Oc			0.01	1.2	0.01	
**Phycidae**								
*Phycis blennoides*	*Pb*	N	0.04	4.8	0.02	3.0	0.02	3.7
*Phycis phycis*	*Pph*	N	0.12	7.9	0.01	2.4	0.05	4.8
**Pleuronectidae**								0.3
*Buglossidium luteum*	*Bl*	N	0.20	9.5	0.51	8.9	0.41	9.2
*Platichthys flesus*		N	0.07	4.0	0.02	1.2	0.04	2.4
Unidentified spp			0.01	0.8			<0.01	
**Scombridae**								
*Scomber scombrus*	*Ss*	N	13.69	66.7	3.23	42.3	6.431	52.7
**Scopelosauridae**								
*Scopelosaurus lepidus*	*Sl*	Oc	0.08	5.6	0.32	20.8	0.25	14.3
**Scophthalmidae**								
*Lepidorhombus boscii*		N	0.02	1.6	0.02	1.8	0.02	1.7
*Phrynorhombus norvegicus*	*Pn*	N	0.20	13.5	0.28	4.2	0.26	8.2
*Phrynorhombus regius*		N	0.04	4.0	0.02	2.4	0.03	3.1
*Scophthalmus rhombus*		N	0.01	0.8			<0.01	0.3
**Scorpaenidae**								
*Helicolenus dactylopterus*		Oc	0.01	0.8	0.01	1.2	0.01	1.0
*Scorpaena porcus*		N			<0.01	0.6	<0.01	0.3
**Serranidae**								
*Serranus cabrilla*	*Sc*	N	0.07	6.3	0.07	8.9	0.07	7.8
**Soleidae**								
*Microchirus variegatus*	*Mv*	N	0.54	19.0	0.47	19.6	0.49	19.4
*Pegusa lascaris*		N			0.01	0.6	0.01	0.3
*Solea solea*		N	0.01	0.8			<0.01	0.3
**Sparidae**								0.3
*Boops boops*	*Bbo*	N	2.91	27.0	10.42	35.7	8.13	32.0
*Diplodus* spp	*D*spp	N	0.04	2.4	0.10	6.0	0.08	4.4
*Spondyliosoma cantharus*	*Sca*	N	0.33	17.5	0.52	15.5	0.46	16.3
Unidentified spp			0.01	0.8			<0.01	
**Sternoptychidae**								
*Argyropelecus hemigymnus*	*Ah*	Oc	0.09	5.6	0.22	19.6	0.18	13.6
*Maurolicus muelleri*	*Mmu*	Oc	2.85	56.3	8.31	73.8	6.65	66.3
**Stomiidae**								
*Stomias boa boa*	*Sb*	Oc	0.98	34.1	0.27	22.0	0.49	27.2
**Sygnathidae**								
*Nerophis lumbriciformis*	*Nl*	N	0.09	6.3	0.14	8.9	0.12	7.8
**Family Trachinidae**								
*Trachinus draco*		N			0.01	1.2	0.01	0.7
*Echiichthys vipera*		N			0.02	2.4	0.02	2.0
**Triglidae**								
*Eutrigla gurnardus*	*Eg*	N	0.04	4.0	0.13	7.7	0.10	6.1
Unidentified sp 3			0.01	0.8	0.01	2.4	0.01	1.7
Unidentified sp 4					0.01	1.2	0.01	0.7
Unidentified sp 5	U*Sp*5				<0.01	0.6	<0.01	3.1
Unidentified sp 12					0.01	0.6	<0.01	0.3
Unidentified sp 13			0.02	1.6	<0.01	0.6	0.01	1.0
Unidentified sp 14			0.14	11.11	0.08	7.7	0.10	9.2
Unidentified spp			1.61	37.3	1.82	44.6	1.76	41.5

The JUREVA 0410 (JC) and both cruises combined (CC + JC); Code: canonical correspondence analysis (CCA) taxon code; Origin (N: neritic; Oc: oceanic); RA: taxon relative abundance (%); %Occ: taxon percentage of occurrence.

Larval fish abundances ranged from 0 to 20,463.45 larvae 10 m^−2^, with an average of 1048.15 (±2186.54 SD) larvae 10 m^−2^, during the CC and from 0 to 30,849.16, with a mean of 1891.62 (±3572.04 SD) larvae 10 m^−2^, during the JC. Differences in larval fish abundances were not significant between cruises, but they were significant among cruises-regions combined, being higher in the SBB during the two cruises (Table 1). The highest larval fish abundances were recorded in the shelf region during both cruises (Fig. 4c,d). However, relatively high abundances were recorded in the oceanic region of the SBB, mainly during the JC, (Fig. 4d).

Individual larval fish species representative of neritic (*S*. *pilchardus* and *Boops boops*) and oceanic (*M*. *muelleri*) species showed different patterns of horizontal distribution. *S*. *pilchardus* showed a wider offshore distribution along the French coast during the CC (Fig. 4e) and along the Spanish coast during the JC (Fig. 4f). *B*. *boops*, which was only collected off the Iberian Peninsula, showed a very coastal distribution during the CC (Fig. 4g) and a wider distribution during the JC (Fig. 4h). *M*. *muelleri* was collected off the shelfbreak during both cruises (Fig. 4i,j).

**Figure 5:**
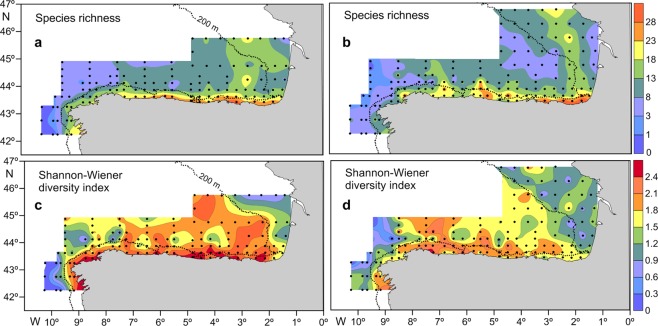
Horizontal distribution of: (**a**) Species richness during the CAREVA cruise (CC); (**b**) species richness during the JUREVA cruise (JC); (**c**) Shannon-Wiener diversity index during the CC; (**d**) Shannon-Wiener diversity index during the JC.

Taxon richness ranged from 0 to 32, with an average of 12.28 (±6.9 SD), during the CC and from 0 to 34, with an average of 12.01 (±7.3 SD), during the JC. Differences in taxon richness were not significant between cruises, but they were significant among cruises-regions combined, being significantly higher in the SBB during both cruises (Table 1). This variable followed similar patterns of horizontal distribution during both cruises (Fig. 5a,b) and was also similar to those followed by larval fish abundances (Fig. 4c,d). In fact, there was a strong correlation between larval fish abundances and taxon richness (Pearson’s correlation coefficient, r = 0.64, p < 0.01).

The values of larval fish diversity recorded during the CC ranged from 0 to 2.67, with a mean of 1.82 (±0.63 SD). During the JC, the values of this ichthyoplankton assemblage parameter ranged from 0 to 2.51, with an average of 1.53 (±0.56 SD). Differences in larval fish diversity were significant between cruises, being higher during the JC, and between regions when the two cruises were combined, being higher in the SBB during both cruises (Table 1). Larval fish diversity showed similar patterns of horizontal distribution during the two cruises (Fig. 5c,d). This variable was positively correlated with larval fish abundance (Pearson’s correlation coefficient, r = 0.30, p < 0.01) and with taxon richness (Pearson’s correlation coefficient, r = 0.63, p < 0.01).

### Larval fish assemblages

The PERMANOVA test revealed significant differences in LFA structure between cruises and among cruises-regions combined (p < 0.01 in both cases). All pairwise comparisons among cruises-regions combined were also significant (p = 0.02 for the region AR sampled during the CC and during the JC; p < 0.01 for all of the other cruises-regions combined pairs). When the two cruises were combined, the difference in assemblage structure between the AR and the SBB was also significant (p < 0.01).

**Figure 6:**
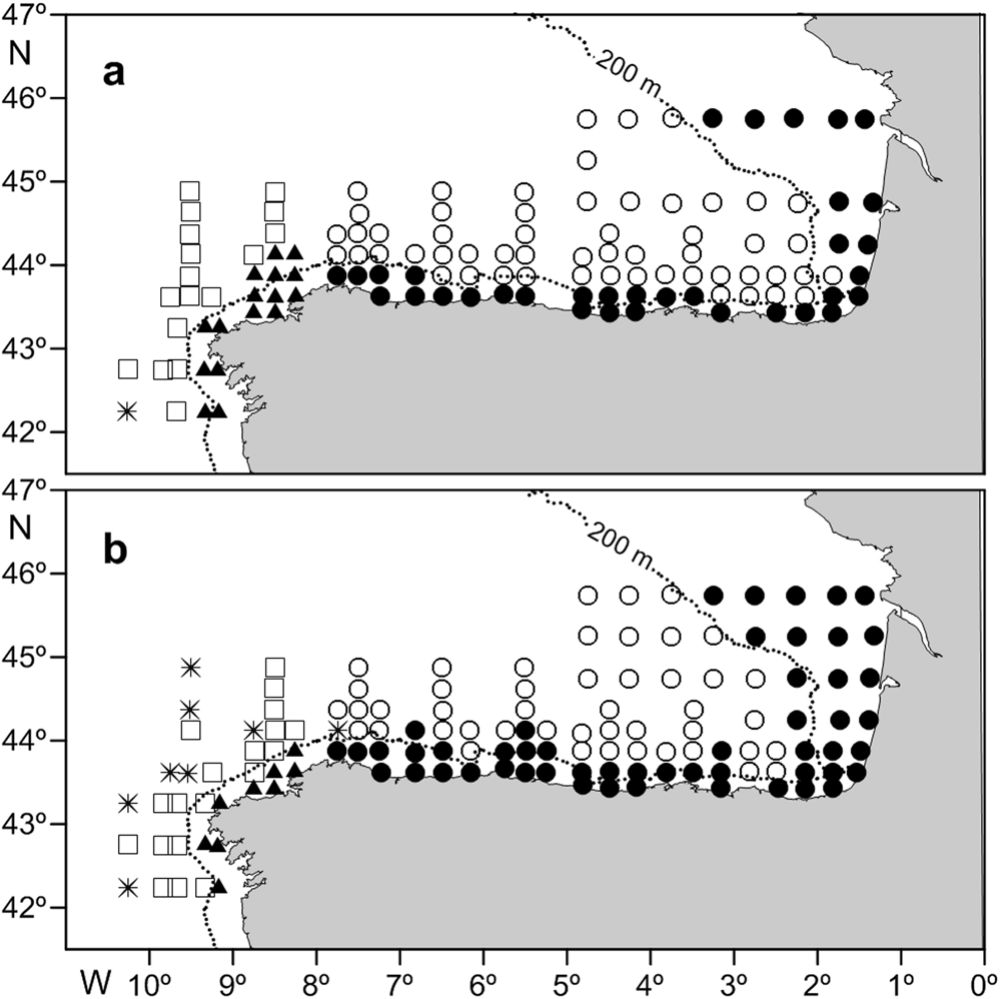
Geographic location of the larval fish assemblages: (**a**) during the CAREVA cruise and (**b**) during the JUREVA cruise, defined by cluster analyses and non-metric multidimensional scaling ordination (nMDS): (▴) shelf assemblages in the Atlantic region (AR); (◽) off-shelf assemblages in the AR; (⚫) shelf assemblages in the southern Bay of Biscay region (SBB); (⚬) off-shef asssemblages in the SBB; () outliers.

Cluster analyses identified two LFAs for each region and cruise at a similarity level of ≤38% (dendrograms not included). The nMDS ordination produced the same results at stress values between 0.13 and 0.16 (nMDS ordination plots not included). Assemblages were named after the geographical location of the sampling stations they occupied, viz. shelf and off-shelf assemblages (Fig. 6a,b). The border between the two assemblages was roughly the 200 m isobath, although during the JC, the shelf assemblage extended further offshore along the SBB coast and showed a more coastal distribution in the AR (Fig. 6b). In this region, the off-shelf assemblage during the JC was not well defined because there were several stations (only one during the CC) located outside the 200 m isobath that were not allocated to either of those two assemblages. These stations were considered outliers (asterisks in Fig. 6a,b).

According to the PERMANOVA tests, differences in structure between the shelf and the off-shelf LFAs were significant for both regions and cruises (p < 0.01 in all cases). The outlier stations were not included in the PERMANOVA analyses.

### Relationship of larval fish taxon distribution with the environmental variables

The environmental variables included in the CCA analysis were depth, sea surface temperature, sea surface salinity and mesozooplankton biomass. Water column stratification was not included in this analysis because of its strong correlation (collinearity) with sea surface salinity during both cruises and with sea surface temperature during the JC. These variables were significant in explaining larval fish taxon distribution (p < 0.01 for both the CC and the JC). However, depth and sea surface temperature were the most important during both cruises.

**Figure 7:**
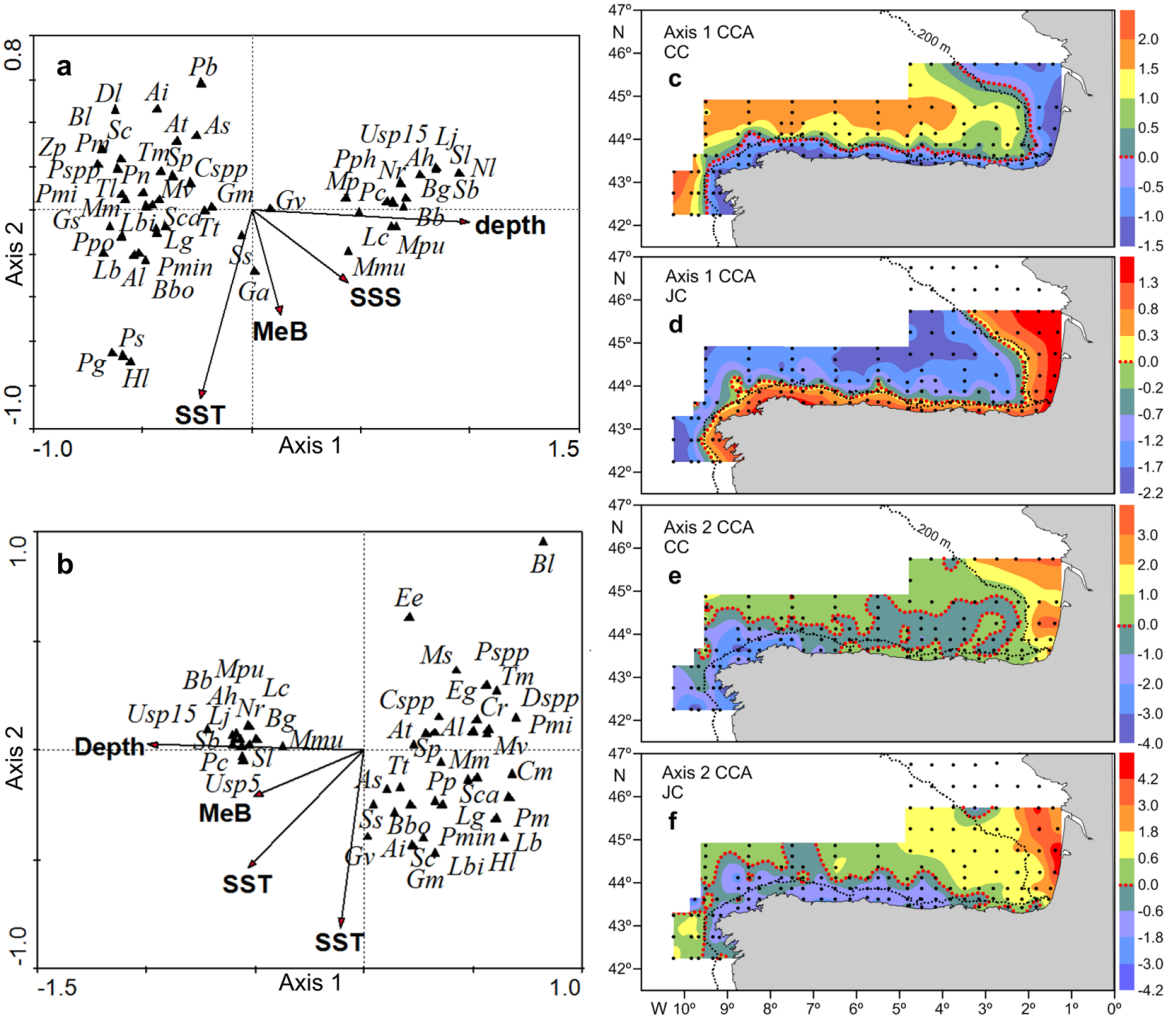
Canonical correspondence analysis (CCA) biplot for environmental variables (arrows) and larval fish taxa (triangles) for: (**a**) CAREVA cruise (CC) and (**b**) JUREVA cruise (JC). Environmental variables were depth (Depth), sea surface temperature (SST, temperature °C at 5 m depth), sea surface salinity (SSS, salinity at 5 m depth) and mesozooplankton biomass (MeB, mg m^−2^). See Table I for larval fish taxon codes. Geographical distribution of CCA axis sample (station) scores for: (**c**) Axis 1 CC; (**d**) Axis 1 JC; (**e**) Axis 2 CC; (**f**) Axis 2 JC. Dotted redlines separate stations with positive and negative station scores. Black dots represent sampling stations.

The first 2 canonical axes (axis 1 and axis 2) were also significant for both the CC and the JC (p < 0.01 in both cases). These axes explained 89.89% (the CC) and 88.22% (the JC) of the variance in the taxon-environment relationship. The taxon-environment correlation coefficients for axes 1 and 2 were 0.93 and 0.76, respectively, for the CC and 0.91 and 0.79, respectively, for the JC. During the CC, depth, sea surface salinity and mesozooplankton biomass were positively correlated with axis 1, while sea surface temperature was negatively correlated with this axis. All variables were negatively correlated with axis 2 (Fig. 7a). During the JC, all variables were negatively correlated with axis 1, and only depth was positively correlated with axis 2 (Fig. 7b). The geographic representation of sample scores on axis 1 indicates that this axis was mainly related to depth for both cruises (Fig. 7c,d). Correspondingly, oceanic taxa were clustered on the right side of the ordination biplots along axis 1 for the CC and on the left side for the JC, with very little vertical dispersion (Fig. 7a,b, respectively). However, neritic taxa showed the opposite pattern, being located on the left side of the ordination biplots for the CC and on the right side for the JC along axis 1, with a wider vertical distribution (Fig. 7a,b, respectively). The geographic representation of sample scores on axis 2 showed that this axis was mainly related to sea surface temperature (Fig. 7e,f).

## Discussion

### Hydrography

According to Fraga^36^, the CC would have been carried out at the time of the transition from the autumn-winter coastal downwelling to the spring-summer upwelling regimes, while the JC would have been carried out when the upwelling regime prevailed. According to the upwelling index values, the transition between the two regimes would have occurred at the end of the CC (Fig. 3). On the other hand, both regimes were reflected by the different offshore extensions shown by the relatively fresh coastal water, mainly related to riverine discharges^23^. During the CC, the Iberian Poleward Current and the surface poleward flow over the shelf, produced by winds favourable to downwelling, would have kept the relatively fresh coastal water close to the coast^22,25^. However, during the JC, the offshore Ekman transport associated with coastal upwelling would have spread fresh surface water farther from the coast^49^, evidenced through the upwelling filaments formed west of the main capes of the region. The large filament located off Cape Finisterre, which affected most of the AR, could have been formed during the strong upwelling event recorded at the end of the CC, because the time response of the system to wind forcing in the region is approximately 3 days and relaxes slowly, so a wind event cycle would span approximately 10 days^50^. Off the French coast, because the largest rivers discharge in the northern part of the study area, the offshore Ekman transport and the shelf equatorward flow associated with coastal upwelling would be responsible for spreading fresh coastal water over the whole French shelf^24,49^.

The relatively salty surface water mass found in most of the oceanic region during both cruises showed values of temperature (>12.2 °C) and salinity (>35.66) characteristic of the warmer and saltier eastern North Atlantic central water of subtropical origin^51^. This water mass is transported by the Iberian Poleward Current, which flows along the Portuguese and Spanish slopes, into the SBB, eventually reaching the French shelf^26,28,29^. The larger area occupied by the warmer and saltier eastern North Atlantic central water of subtropical origin during the JC could have been the result of the strong downwelling recorded during most of the CC. Downwelling events are followed by intense surface poleward flow, and they are also apparently related to the strong intrusion of the Iberian Poleward Current into the Bay of Biscay^52^. The zonal gradient of sea surface temperature during the CC supports the Iberian Poleward Current intrusion into de Bay of Biscay. Sea surface temperature, because of surface cooling, diminishes eastward, as the warmer and saltier eastern North Atlantic central water of subtropical origin progresses in this direction. The border between the fresher coastal waters and the haline oceanic waters located around the shelfbreak constituted the convergent front associated with the Iberian Poleward Current, as reported by Lavin *et al*.^22^ and Fernandez *et al*.^30^, during the CC and with the upwelling front associated with any coastal upwelling^34^, during the JC. The shelf-break front was disrupted by the upwelling filament related to Cape Finisterre during the JC, as revealed by the offshore extension of fresher coastal waters (Fig. 2d). Therefore, the shelf-break front observed in this study seems to be a permanent hydrographic feature of the region during the downwelling season, but it only seems to remain in the SBB during the upwelling season.

The higher sea surface temperature found during the JC is consistent with the sea surface temperature increase as the spring progresses. In turn, the absence of significant differences in sea surface salinity between the CC and the JC indicates that the region was occupied by the same surface water mass during both cruises. Additionally, the higher stratification found during this cruise could be related to the temperature increase in the surface layers of the water column. However, the water column stratification was mainly determined by salinity, as it indicates the strong correlation found between sea surface salinity and the stratification index.

### The ichthyoplankton assemblage

The distribution of fish eggs among species reveals the succession in the spawning peak of the species that dominate the pelagic fish assemblage in the area of study: *S*. *scombrus* and *S*. *pilchardus*^53,54^. Thus, *S*. *scombrus* spawning peaks at the time of the CC^55,56^, while *S*. *pilchardus* spawning peaks at the time of the JC^57,58^.

The overall species composition and structure of the LFA roughly reflected the composition and structure of the adult fish assemblage inhabiting the shelf-slope region in the area of study^54,59^. In this way, the LFA was dominated by *S*. *pilchardus*, the most abundant pelagic fish species in the region, at the time of the study^60^. *B boops* and *S*. *scombrus*, the second and the fourth most abundant larval fish species, were also identified as top-ranking species in the adult fish assemblage in our study area by Santos *et al*.^54^ and Fariña *et al*.^59^. The most noticeable differences between the larval and adult fish assemblages were the relatively low abundances of *Micromesistius poutassou* larvae, the most abundant demersal fish species in the region^59^, the relatively high larval abundances of the mesopelagic species *M*. *muelleri* and the relatively large number of oceanic larval fish species. *M*. *poutassou* was only relatively abundant during the CC, which occurred at the end of the spawning period of this species in the region^61,62^. The difference in the on shelf-off shelf extension of the area sampled by Santos *et al*.^54^ and Fariña *et al*.^59^, which was restricted to the shelf and upper slope, and the area sampled in this study, with most of the sampling stations located off the shelf break, would account for the relatively high number of oceanic larval fish species and for the relatively high abundance of *M*. *muelleri* larvae recorded in this study. Therefore, the similarities between the adult and larval fish assemblages found in this study, carried out when most of the fish species reproduce in the region^61–63^, support the use of ichthyoplankton surveys as a system to monitor fish communities and their response to changing ocean conditions^19^. This should encourage marine research institutions devoted to fisheries research and management to use routine ichthyoplankton samplings aimed towards obtaining information on a single or a few species to also use such sampling methods to monitor the response of fish communities to global warming.

The higher ichthyoplankton abundance, larval fish taxon richness and diversity found in the SBB indicate that the ichthyoplankton assemblage in this region was more abundant and diverse than that in the AR. This supports the Bay of Biscay as a distinct marine ecosystem^21^ and suggests that the SBB meets the conditions underlying favourable reproductive habitats for marine fishes: nutrient enrichment, prey concentration and larval fish retention^64^. In the shelf region, river discharges and coastal upwelling may account for nutrient enrichment^32,39^, and the shelf-break front may account for the prey concentration^30^ and for the retention of the larvae of neritic fish species, as discussed below. The dynamics of SWODDIES, which form in the SSB, produces vertical transport of nutrients into the euphotic layer and an increase in the primary production, chlorophyll a concentration and mesozooplankton biomass within these eddies^29,39,65^. The patch of relatively high mesozooplankton biomass found in the oceanic region of the SBB during the JC (Fig. 2h) could be related to these mesoscale structures. Thus, SWODDIES would also meet conditions underlying the favourable reproductive habitats for oceanic fish species. This is supported by the relatively high abundances of fish offspring recorded in the oceanic region of the SBB, fish eggs during the CC and fish larvae, especially during the JC. Eddies may also trap, concentrate and transport zooplankton organisms, including fish larvae^2,66,67^. It has been suggested that SWODDIES trap *Merluccius merluccius* larvae and transport them towards their recruitment areas when these eddies move close to the coast or over the continental shelf, aiding in recruitment^68^. The SBB, apart from constituting a favourable reproductive habitat for marine fishes, definitely seems to meet favourable conditions for the replenishment of neritic fish populations because even those neritic larvae that drift beyond the shelf-break front could be trapped by the SWODDIES, where they could find appropriate conditions for their development and return to the neritic region transported by these eddies when they approach the coast. The functioning of eddies as nursery areas has already been suggested^2,67^.

### Larval fish assemblages

The difference in LFA structure between the AR and the SBB indicates that these two regions were inhabited by different LFAs, which adds support to the recognition of the Bay of Biscay as a distinct ecosystem. Additionally, this difference was the only significant alongshore variability in the LFA structure. The strongest variability is generally found in the inshore-offshore direction, with assemblages paralleling the coast^44,69–71^. On narrow shelves, the presence of only two assemblages has been widely reported around the world^69,71,72^ and even in our area of study^10,11^. The presence of assemblages paralleling the coast suggests depth as being the most important environmental variable shaping the cross-shelf structure of the LFA. This was supported by the CCA results, which identified depth as being the most important variable explaining larval fish species distributions. However, during both cruises, the shelf assemblages in the SBB were restricted to the region occupied by fresher coastal water, while the off-shelf assemblages were limited to the slope-oceanic region, and the boundary between them was roughly defined by the shelf-break front, which was located further offshore during the JC. This indicates that this front in this region acted as a barrier for both the offshore dispersal of fish larvae of neritic species, supported by the correspondence of the offshore limit of the distribution of larvae of fish species with coastal spawning (e.g., *S*. *pilchardus* and *B*. *boops*) with the shelf-break front, and the shelf intrusion of larvae of oceanic species, supported by the restriction of the slope-spawning species *M*. *muelleri* to the oceanic region. The functioning of the shelf-break front in the SSB as a barrier, maintaining neritic fish larvae on the coastal side of the front and oceanic fish larvae on the oceanic side, has already been reported^10,30^. In the AR, the situation was significantly different because during the JC, under conditions of coastal upwelling, the coastal assemblage showed a more coastal distribution than that during the CC, which was carried out under a regime of coastal downwelling. This suggests that larval fish distributions are good indicators of downwelling and weak upwelling regimes that do not disrupt the shelf break front but not of relatively strong upwelling or upwelling filaments that disrupt the shelf break front. The latter may be related to the different spawning strategies and larval behaviour exhibited by neritic fish species in regions with strong coastal upwelling to avoid or minimize the offshore loss of their offspring through Ekman transport^3,73,74^. Rodriguez *et al*.^75^, in a study carried out in the AR during the late winter mixing period with the water column completely mixed, a situation similar to that found in this study, reported an average depth distribution of 56.6 m for fish larvae. A relatively deep distribution of fish larvae in the water column would place larvae in the subsurface offshore (onshore) flow associated with coastal downwelling (upwelling), accounting for the different onshore-offshore extension of the coastal assemblage during the two cuisses.

The other important factor in the larval fish species distribution was sea surface temperature. The small dispersion of the oceanic species along axis 2, mainly related to sea surface temperature, in the CCA biplots is related to the minor sea surface temperature variability in the oceanic region. Conversely, the larger dispersion of neritic species along this axis is related to the higher sea surface temperature variability in the shelf region.

In summary, this is the first study that, using opportunistic ichthyoplankton sampling, addresses the ichthyoplankton assemblages of the shelf and oceanic regions of the AR and the SBB. The hydrographic situation was of coastal downwelling during most of the CC, except at the end of this cruise, when a relatively strong upwelling event was recorded, and of weak coastal upwelling during the JC, and the transition between the two hydrographic regimes occurred at the end of the CC. The most relevant hydrographic feature found was the shelf-slope front, which was present during both cruises in the SBB and was disrupted by an upwelling filament in the AR during the JC. The ichthyoplankton assemblage was dominated by *S*. *scombrus* eggs and *S*. *pilchardus* larvae, and these species also dominated the pelagic fish community in the area at the time of the study. In both regions, the LFA was structured into on-shelf and off-shelf assemblages independent of the hydrographic situation. The results of this study support the Bay of Biscay as being a distinct marine ecosystem and its southern region, inhabited by a richer ichthyoplankton assemblage, as being a favourable spawning ground and nursery area for the fish assemblage of the region. These results should also encourage marine research institutions to use routine ichthyoplankton sampling to monitor fish communities and their response to global warming.
